# Supplementary material for: Transcranial direct current stimulation and working memory: Comparison of effect on learning shapes and English letters
Source: PLoS One. 2020 Jul 24;15(7):e0222688. doi: 10.1371/journal.pone.0222688 (PMC7380606; doi:10.1371/journal.pone.0222688)
Supplement: Attachment — Submitted filename: Response to Reviewers.docx [file pone.0222688.s002.docx]

Response to Reviewer’s Comments

We thank the reviewer for the overall positive appraisal of the quality of the paper and the constructive feedback given. We are pleased to note that the reviewer found our paper technically sound, the data support the conclusion, the statistical analysis was appropriate and rigorous and the manuscript was intelligible and of good writing standard.

Below, we show how we have addressed all the comments made by the reviewer and revised the paper accordingly. We have corrected all the errors and typos, added explanations as requested and clarified a couple points around methods. We believe the reviewers’ feedback has helped increased the quality and readability of the paper.

**Reviewer #1:**

**The authors employed twenty males to investigate the effect of A-tDCS on the left DLPFC when doing a working memory task (i.e., 2-back task, shape or letter). They stated that a significant A-tDCS effect was found in the shape working memory task not in the letter task. But the following issues should be addressed.**

**1. Participant Selection. More information should be provided (e.g., handness, and vision).**

**Authors:** We have taken on board this comment and have addressed it in Page 4, line 4. All the participants were right handed.

**2. Working Memory Measurement Protocol. More details should be provided to improve its repeatability. When did the participants need to make a response? the onset of shape/letter? or the 2s-blank? Did you ask the participants to response as good and fast as possible?**

**Authors:** The aim of this study was not to measure the reaction time, so participants were told to respond anytime from the cue is displayed to the end of the 1.5s-blank period (before the starting of next cue). This is now explained in page 5, line 26-27.

**2.1 How about the practice phase before the formal test?**

**Authors:** Yes, we can confirm that every participant had a practice test (the sequence of random shapes/alphabets used in the practice are different to that of sequence used in experiment). This has been clarified now in page 5, line 31-33.

**2.2 One task had only one block? How long would the formal experiment take?**

**Authors:** Each block contains two runs, each run consists of 50 cues, with each cue displayed for 2s, and an inter-cue interval (inter-stimulus interval) of 1.5s. This adds up to 50*2+49*1.5=173.5s (~3mins). After the first run, the subjects were given a recovery time of 15s followed by second run. Total time for a block: 3min+3min+15s= 6min 15s.

The above explanation has now been added to the Methods section (page 5, line 34-38).

**2.3 How did you carry out “double-blinded”? Make these clear.**

**Authors:** The below paragraph was already present in the original manuscript (Page 4, line 31-37)

*“An independent investigator gave two conditions of codes (determining the type of stimulation) to the researcher conducting the experiment who was unaware of which code was active tDCS. The researcher then entered the codes into the tDCS unit for each experiment, which was then performed. The second session was conducted using a complementary code so that each participant underwent one sham and one tDCS session without either the participant or the researcher knowing which one was which. “*

The above paragraph explains the process of double blinding where neither researcher nor participants knew which stimulation (real or sham) is being applied.

**3. Results. More analyses should be added and corrected. The authors should add a separate part to describe the data analysis, not in the Results section.**

**Authors:** We have now added a “Statistical Analysis” section above the “Results” section in Manuscript.

**3.1 What’s the meaning of ‘TP’…in the Function 1?**

**Authors:** This comment has now been addressed in Page 6, line 13.

**3.2 Please provide mean and SD/SE values in each condition.**

**Authors:** This comment has now been addressed in Page 6, line 16-17.

**3.3 Add effect size (partial eta square) after p value when doing ANOVA.**

**Authors:** Partial eta square has now been added after p-value in two-way ANOVA results (page 6, line 33-36) as advised by the reviewer.

**3.4 F(1,76,)should be F(1,76).**

**Authors:** This comment has now been addressed in Page 6, line 33.

**3.5 Why not report the results of RT that is also important for n-back task? I expect to see the results of RT in the revision.**

**Authors:** Response time, though we agree is an important topic, was not reported because it was not the subject of the investigation, which was strictly focused on recall accuracy. The data around recall time was not retrievable and unfortunately could not be added retrospectively.

There are precedents in published work around n-back test and tDCS not focusing on response time such as the papers below.

- Mylius, V., Jung, M., Menzler, K., Haag, A., Khader, P.H., Oertel, W.H., Rosenow, F. and Lefaucheur, J.P., 2012. Effects of transcranial direct current stimulation on pain perception and working memory. *European journal of pain*, *16*(7), pp.974-982.
- Cheng, C.P., Chan, S.S., Mak, A.D., Chan, W.C., Cheng, S.T., Shi, L., Wang, D. and Lam, L.C.W., 2015. Would transcranial direct current stimulation (tDCS) enhance the effects of working memory training in older adults with mild neurocognitive disorder due to Alzheimer’s disease: study protocol for a randomized controlled trial. *Trials*, *16*(1), p.479.

**3.6 I find one subject’s data is abnormal (below the chance level of 0.5) in the Fig 2A. Please provide the other version of the statistical results in the revision where you should delete this data point to find out if it affects the current results.**

**Authors:** The Y-axis in Figure 2A is refers to accuracy scores (not probabilities. We are happy to provide the results without this subject.

The results are recomputed after removing the outlier subject using Two-way ANOVA results are as follows:

- Stimulation (sham and real): F(1,76)=3.91, *p*=0.05, partial eta=0.05
- Stimuli (letters and alphabets): F(1,76)=5.17, *p*=0.02, partial eta=0.06
- Interaction effect: F(1,76)=0.068, *p*=0.8, partial eta=0.001
- Two-tail paired t-test between sham and tDCS across shapes: 0.02 (Cohen’s *d*=0.56)
- Two-tail paired t-test between sham and tDCS across letters: 0.15 (Cohen’s *d*=0.36)

We have presented the non-altered data and altered data (after removing outlier) in the paper for completeness.

**3.7 The interaction effect was not significant, so why did you conduct a simple effect analysis by t-test? This doing seems not follow statistical requirements.**

**Authors:** Our understanding is, if there is a significant interaction effect, then the post-hoc on the main effects are often not of interest. However, the post-hoc on the interaction is of interest.

**But if there isn’t** a significant interaction effect, but there **are** significant main effects, then the post-hoc is performed on the significant main effects.

A number of papers indicate this approach: Wei et al (“Comparisons of treatment means when factors do not interact in two-factorial studies”, Amino Acids, 2012 42(5):2031-5) provide examples of 2-factor studies where it is useful to perform a post-hoc analysis when only one factor and not both factors or their interaction is significant. In the abstract, they wrote "*when the two factors do not interact, a common understanding among biologists is that comparisons among treatment means cannot or should not be made. Here, we bring this misconception into the attention of researchers.* "

**3.8 The df and t values are wrong (t(38)=-2.81, p=0.011...).**

**Authors:** We have now addressed this comment in page 7, line 21.

**4.1 . Discussion. Not using 0-back as the control condition should be the other one limitation. As far as know, 0-back is the control condition for the classical n-back task. Why not use it in your study? Else, please discuss the influence of task difficulty between these two tests on your results.**

**Authors:** Our understanding is 0-back test means no recall involved/comparison between current cue and past ones in n steps back stimuli (if n=0). We can see how this can be used as a baseline to ensure participants are at least able to operate the experiment (click when they see the cues).

N-back test as a working memory is obviously useful from n=1 and higher. We did experiment in pilot experiment, with values of n and yes, test difficulty increases with increase in n value. We looked at the literature the highest “n” used to the authors knowledge (Ohn eta l., 2007; Frengi et al., 2005; Nilson et al., 2015) is 3 and lowest is 1 (Jonides et al., 1997; Ragland eta l., 2002; Carlson et al., 1998).

Prior to starting this experiment, the question of what is the appropriate value for n was asked, and the team did run a smaller pilot with n values (1, 2 and 3). For n=1 accuracy was close to 100% (too easy) , whereas n=3 was difficult for subjects with accuracy mostly close to 0% (too hard). 2-back was found to provide the most range of accuracy results and hence was chosen for the main experiment.

We have now added the above statement in the methods section (page 6, line 4-7).

**5.1 Reference. The order of 54 and 55 is wrong.**

**Authors:** Unless we are misunderstanding the reviewer’s comment, the order seems to be correct. Ref 54 talks about repeated A-tdcs sessions and Ref 55 talks about steroid hormones effecting the mood and concentration level.

**6. Figure 1. The rectangle in the top left corner of Fig 1 seems to be unnecessary. The red line under the “tDCS” should be removed.**

**Author:** This comment has now been addressed.

We thank again the reviewer for his/her time examining our manuscript, and we remain happy to give any further details and clarification if needed before the final publication of the paper.
