# Supplementary material for: Transcranial direct current stimulation and working memory: Comparison of effect on learning shapes and English letters
Source: PLoS One. 2020 Jul 24;15(7):e0222688. doi: 10.1371/journal.pone.0222688 (PMC7380606; doi:10.1371/journal.pone.0222688)
Supplement: Attachment — Submitted filename: Response to Reviewers.docx [file pone.0222688.s003.docx]

# **Response to Reviewers comments**

**Reviewer #1: The author has responded to and revised some of the comments. However, I still think that some problems have not been directly explained by the authors. First, there is a lack of baseline condition (0-back). Second, no RT results were presented. Both of these are very important for experimental design and consequence inference.**

We thank the reviewer again for their constructive comments and for acknowledging that most issues have been addressed with the exception of only two remaining questions, around use of 0-back test and, the reaction time measurement which we accept are fair, and legitimate points and shall attempt to address below.

0-back test:

Upon further consideration of this concept, we understand the reviewer’s point as is referring to a baseline condition indicating that the subjects recognised the presence of an appropriate symbol and then reacted with no demand on memory, as in the reference below:

"In the 0-back condition, the target was any letter that matched a pre-specified letter (i.e., “c”). **Thus, this condition required sustained attention but no working memory demand."** p.712 (Miller et al, 2009)

In this case, this was performed, though admittedly not a formal part of the study, as part of the pre-study pilot we referred to in the paper were subjects were asked to accustom themselves to the test and as part of that process, respond to visual letter stimuli presented. The accuracy rate for that was 100%. We did not label it as a 0-back test as it was seen as simply testing that baseline condition and ensuring subject were “capable” of performing the main 2-back test. We did not formally test the sustained attention element, however as each test period on a single stimulus type was circa 3 minutes, attrition was not considered an important factor.

We have now added this important clarification to the paper in (page 6-line 4).

As we mentioned in our previous response we went further in the pilot, performing the 1-back and 3back tests in the pilot; however decided not to pursue these tests for the main experiment as the dynamic variation for each was small (all results being near 100% or near 0% accuracy, for the 1-back and 3 back respectively).

Reaction time:

Here also, we understand the important of response time in research aimed at studying short term memory. We agree with the reviewer that RT is essential before drawing any conclusions about mechanisms of any reported effects on the memory. We have added this line to our paper Page 8, Line 39 when discussion the limitations of the study:

“Finally, the study did not look at “reaction time” and its interaction with accuracy and whether a ceiling effect (Hur et. al. 2017) could have been affected by tDCS application.”

This being said, none inclusion of response time appears to be common when the target of study is not to understanding memory per say but the effect of a factor (in our case tDCS) on accuracy specifically. Examples of recent such peer reviewed research work below are

- Scharinger, C., Soutschek, A., Schubert, T. and Gerjets, P., 2017. Comparison of the working memory load in n-back and working memory span tasks by means of EEG frequency band power and P300 amplitude. *Frontiers in human neuroscience*, *11*, p.6.
- Soveri, A., Antfolk, J., Karlsson, L., Salo, B. and Laine, M., 2017. Working memory training revisited: A multi-level meta-analysis of n-back training studies. *Psychonomic bulletin & review*, *24*(4), pp.1077-1096.
- Yaple, Z. and Arsalidou, M., 2018. N‐back working memory task: Meta‐analysis of normative fMRI studies with children. *Child development*, *89*(6), pp.2010-2022.

We hope our acknowledgment of the RT limitation in the paper, along with the fact paper is not at complete odds with similar peer reviewed research, will meet the reviewer’s, if not full satisfaction, minimal approval for accepting this work.

Best regards,

Authoring teams
