# Supplementary material for: Transcranial direct current stimulation and working memory: Comparison of effect on learning shapes and English letters
Source: PLoS One. 2020 Jul 24;15(7):e0222688. doi: 10.1371/journal.pone.0222688 (PMC7380606; doi:10.1371/journal.pone.0222688)
Supplement: Attachment — Submitted filename: Response to Reviewers_ThirdRound-Final.docx [file pone.0222688.s004.docx]

# **Response to Reviewers comments**

**Reviewer #1: Minor edit to address before endorsing publication:
The df of t tests in Page 7, Lines 20 and 21 should be 19. Please check and modify it.**

We have now addressed the final comment of the reviewer. The updated values can be found page 7, lines 15 and 16.

We thank the reviewer again for their constructive comments which have invariably improved the quality of our paper.
